# Of microbes and mange: consistent changes in the skin microbiome of three canid species infected with *Sarcoptes scabiei* mites

**DOI:** 10.1186/s13071-019-3724-0

**Published:** 2019-10-16

**Authors:** Alexandra L. DeCandia, Kennedy N. Leverett, Bridgett M. vonHoldt

**Affiliations:** Department of Ecology & Evolutionary Biology, Princeton University, Princeton, NJ USA

**Keywords:** Microbiome, Dysbiosis, Sarcoptic mange, *Sarcoptes scabiei*, Coyotes, Red foxes, Gray foxes, Secondary bacterial infection, *Staphylococcus pseudintermedius*, *Corynebacterium*

## Abstract

**Background:**

Sarcoptic mange is a highly contagious skin disease caused by the ectoparasitic mite *Sarcoptes scabiei*. Although it afflicts over 100 mammal species worldwide, sarcoptic mange remains a disease obscured by variability at the individual, population and species levels. Amid this variability, it is critical to identify consistent drivers of morbidity, particularly at the skin barrier.

**Methods:**

Using culture-independent next generation sequencing, we characterized the skin microbiome of three species of North American canids: coyotes (*Canis latrans*), red foxes (*Vulpes vulpes*) and gray foxes (*Urocyon cinereoargenteus*). We compared alpha and beta diversity between mange-infected and uninfected canids using the Kruskal–Wallis test and multivariate analysis of variance with permutation. We used analysis of composition of microbes and gneiss balances to perform differential abundance testing between infection groups.

**Results:**

We found remarkably consistent signatures of microbial dysbiosis associated with mange infection. Across genera, mange-infected canids exhibited reduced microbial diversity, altered community composition and increased abundance of opportunistic pathogens. The primary bacteria comprising secondary infections were *Staphylococcus pseudintermedius*, previously associated with canid ear and skin infections, and *Corynebacterium* spp., previously found among the gut flora of *S. scabiei* mites and hematophagous arthropods.

**Conclusions:**

This evidence suggests that sarcoptic mange infection consistently alters the canid skin microbiome and facilitates secondary bacterial infection, as seen in humans and other mammals infected with *S. scabiei* mites. These results provide valuable insights into the pathogenesis of mange at the skin barrier of North American canids and can inspire novel treatment strategies. By adopting a “One Health” framework that considers mites, microbes and the potential for interspecies transmission, we can better elucidate the patterns and processes underlying this ubiquitous and enigmatic disease.
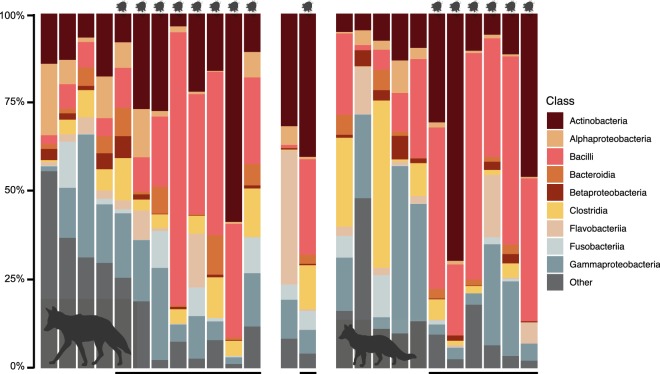

## Background

Sarcoptic mange has been termed a “ubiquitous neglected disease” [1, 2]. Although it afflicts over 100 mammal species on every continent except for Antarctica, numerous questions remain about its pathology [3–6]. A major impediment regards the wide-scale variability that sarcoptic mange exhibits at every level of infection from individuals to populations to species, despite its universal source being *Sarcoptes scabiei* mites [7].

Canids typify this variation. Considered prominent hosts of mange, many canid species are particularly susceptible due to their den usage and sociality [3, 8, 9]. Yet individuals are not affected uniformly. Host symptoms range from mild pruritus to emaciation, dehydration, crust formation or even death [4, 10–12]. This variation scales to the population and species levels, where sarcoptic mange can exist as an enzootic parasite that imposes persistent, low levels of morbidity, or an epizootic parasite that causes dramatic mortality events in virulent outbreaks [8, 13–20].

Amid this variability, it is important to elucidate consistent drivers of morbidity, particularly at the skin barrier. Considered the first line of defense against infection, the skin presents a physical and microbial barrier to invading parasites [21–23]. Upon contact with this barrier, adult females burrow into the skin to feed on host lymph and deposit the next generation of eggs [1, 2]. Often completing their entire life-cycle on the same host, mites and their secretions continuously irritate the skin and elicit severe allergic reactions [3, 4, 6]. Secondary bacterial infection with pathogenic microbes (such as *Staphylococcus* spp. and *Streptococcus* spp.) typically follows mite infestation [6, 24]. Mites may even facilitate colonization of opportunistic invaders by transporting harmful bacteria to the host’s skin [25] and secreting immune inhibitors into burrows and lesions [26, 27].

To examine the effect of sarcoptic mange on the skin microbiome, Swe et al. [28] experimentally infected pigs (*Sus scrofa domesticus*) with *S. scabiei* var. *suis* and sequenced microbial communities over the course of infection. Mange-infected individuals displayed lower levels of microbial diversity, altered community abundance and increased incidence of *Staphylococcus* spp. compared to their uninfected counterparts. Similar patterns have been observed in humans, domestic animals and wildlife infected with sarcoptic mange [1, 6, 12, 29], as well as domestic dogs (*Canis familiaris*) and humans with allergic skin conditions, such as atopic dermatitis [30–34]. This evidence suggests that disrupted microbial communities may play a key role in the pathogenesis of sarcoptic mange.

Given the pervasive variability of this neglected disease, additional studies are needed to assess the universality of these trends. We contributed to these efforts by characterizing the skin microbiome of mange infection across three species of North American canids: coyotes (*Canis latrans*), red foxes (*Vulpes vulpes*) and gray foxes (*Urocyon cinereoargenteus*). Canids present an ideal system for these analyses, as they are among the primary species affected by sarcoptic mange in North America [20]. Due to the divergent evolutionary histories of these three genera, we anticipated species-specific differences in microbial community composition of healthy and infected individuals. However, given their similar ecologies, we predicted consistent responses to mange infection that included decreased species richness and altered community abundance favoring pathogenic bacteria.

## Methods

### Sample and data collection

We opportunistically collected samples from coyotes, red foxes and gray foxes admitted to licensed wildlife rehabilitation centers between January 2017 and April 2019. Partnering centers included the Wildlife Rehabilitation Center of Minnesota (Minnesota), Fund for Animals Wildlife Center (California), Janet L. Swanson Wildlife Health Center at Cornell University (New York), Woodlands Wildlife Refuge (New Jersey), PAWS Wildlife Center (Washington) and Tufts Wildlife Clinic (Massachusetts). Critically, samples were collected upon admission to each facility and before treatment with antimicrobials, antivirals, anthelmintics or acaricides. This minimized potential confounding effects of artificial environment (such as indoor facilities or human contact), sampling location or treatment regime.

Sample metadata included sampling date and location, primary reason for admission, species, sex, age, weight and mange status. We assessed mange severity by assigning each individual to a mange class corresponding to the percentage body area that exhibited symptoms, such as lesions, crusts or alopecia. Uninfected individuals were assigned to Mange Class 0, with Mange Class 1 defined as 0–5% of the body covered, Mange Class 2 by 6–50% and Mange Class 3 by more than 50%, following [35].

**Fig. 1:**
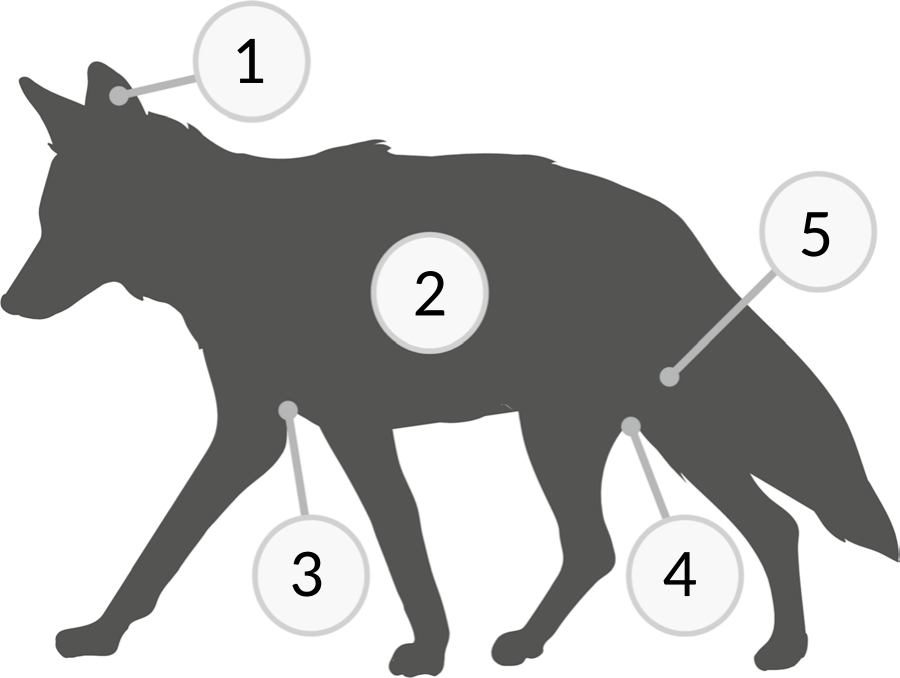
The five body sites swabbed included: (1) external ear, (2) dorsal flank, (3) axilla, (4) groin, and (5) outer back leg. Figure created with BioRender

We collected swabs from five body sites (Fig. 1) that included the external ear, dorsal flank, axilla, groin, and outer back leg. We used a sterile BBL™ swab to sample the skin at each body site, rotating the swab tip by 90° every 10 strokes for a total of 40 swab strokes [30]. Samples were stored at − 80 °C until DNA extraction.

### DNA extraction and *16S* rRNA V4 sequencing

We extracted microbial DNA from each swab tip using a modified DNeasy PowerSoil Kit (Qiagen, Hilden, Germany) protocol described in DeCandia et al. [36]. Briefly, we placed each swab tip into a PowerBead tube and used a TissueLyser II (Qiagen, Hilden, Germany) to disrupt samples for two cycles, both 12 min at 20 shakes/s, with the addition of 60 μl of C1 solution in-between cycles. For the final elution step, we incubated samples at room temperature for 10–15 min using 60 μl of C6 solution pre-heated to 70 °C. We used sterile swab tips as negative controls during every round of extractions to minimize contamination risk. We subsequently concentrated extracts to 20 μl in a Vacufuge and assessed DNA concentrations using a high-sensitivity Qubit™ fluorometer. We used molecular grade water to standardize samples to 2.5 ng/μl and included low yield samples in subsequent steps.

We amplified and tagged the *16S* ribosomal RNA (rRNA) hypervariable 4 (V4) region in each sample through polymerase chain reaction (PCR) using 96 unique combinations of barcoded forward (*n* = 8) and reverse (*n* = 12) primers [37]. As in DeCandia et al. [36], the reaction recipe included 5 μl of HiFi HotStart ReadyMix (KAPA Biosystems, Wilmington, USA), 3.2 μl of primer mix (1.25 μM) and 1.8 μl of template DNA. Cycling conditions were as follows: initial denaturation at 94 °C for 3 min; touchdown cycling for 30 cycles of 94 °C for 45 s, 80–50 °C for 60 s, 72 °C for 90 s, decreasing 1 °C each cycle; 12 cycles of 94 °C for 45 s, 50 °C for 60 s, 72 °C for 90 s; and final extension of 72 °C for 10 min. We used Quant-iT™ PicoGreen™ dsDNA assays (Invitrogen, Carlsbad, USA) to quantify the PCR products, pooled equal nanograms of each library and selected for amplicons between 300 and 400 nt in length using Agencourt AMPure XP magnetic beads (Beckman Coulter, Brea, USA). We sent final libraries to the Princeton University Genomics Core Facility for paired-end amplicon sequencing (2×150 nt) on an Illumina MiSeq machine (Illumina, San Diego, USA).

### Data processing

We used a paired-end, dual-indexed barcode splitter implemented in *Galaxy* to demultiplex raw sequencing data, allowing for one nucleotide mismatch between expected and observed barcode sequences [38]. We then imported reads into *QIIME 2* v.2019.4 [39, 40] for data filtering. Through the *dada2 denoise-paired* plugin, we corrected probable sequencing errors, removed chimeras, trimmed low quality bases and merged paired-end reads to identify taxonomic features [41]. We additionally identified operational taxonomic units (OTUs) using *de novo-*, *closed reference-* and *open reference* clustering with *qiime vsearch* to compare our denoised dataset to more traditional cluster-based methods [42].

### Alpha and beta diversity

We calculated alpha and beta diversity metrics using the *core-metrics-phylogenetic* and *alpha-rarefaction* functions in *QIIME 2*. To correct for differences in read depth, we rarefied samples to 5153 sequences for the full dataset (*n* = 125 samples) and 17,693 sequences for a composite dataset where samples were grouped by individual (*n* = 25 grouped samples). Read depths were chosen to retain all samples for analysis.

To examine within-sample diversity, we calculated the Chao 1 index for species richness and Pielou’s evenness metric for species equitability. For between-sample differences, we used *fasttree* to construct a rooted phylogenetic tree of taxonomic features and calculated unweighted UniFrac distances for species presence, weighted UniFrac distances for species presence and abundance, and the Bray-Curtis dissimilarity index for species abundance. We visualized sample dissimilarity through principal coordinates analysis (PCoA) using the *EMPeror* plugin [43] and performed significance testing using the Kruskal–Wallis test for alpha diversity metrics and multivariate analysis of variance with permutation (PERMANOVA) for beta diversity differences [44]. Variables of interest included sampling state, species, age, sex, year and mange infection status.

### Taxonomic composition and differential abundance testing

We determined the taxonomic composition of each sample using a Naïve Bayes classifier trained on Greengenes 13_8 reference sequences trimmed to our *16S* rRNA V4 amplicon and clustered at 99% similarity [45, 46]. We then used the *classify-sklearn* function to assign taxonomy to each representative sequence in the dataset [46].

To assess the statistical significance of compositional differences, we used two complementary approaches for differential abundance testing: analysis of composition of microbes (ANCOM) and gneiss balances. ANCOM calculates the log-ratio between pairwise combinations of taxa and sums how many times the null hypothesis is violated [47]. Gneiss calculates log transformed ratios (termed balances) between groups of taxa arranged in a hierarchical tree through correlation clustering [48]. Ordinary least squares (OLS) regression can subsequently be used to test for differences between infection groups. Both analyses require a composition artifact as input, with additional filtering necessary to remove taxonomic features that occur in fewer than 10 samples or have frequencies below 50. We implemented each analysis with our composite dataset where samples were grouped by individual, and queried results using the NCBI BLASTn online tool [49].

## Results

### Amplicon sequencing and data processing

We sequenced 153 samples collected from 15 coyotes (mange-infected = 9, uninfected = 5, unknown = 1), 13 red foxes (mange-infected = 8, uninfected = 5) and 2 gray foxes (mange-infected = 1, uninfected = 1). The full dataset contained 4,397,629 raw reads, which reduced to 3,911,712 sequences after denoising (Additional file 1: Table S1). The denoised dataset contained 11,800 unique taxonomic features, whereas the OTU datasets contained 6137 (*de novo)*, 5456 (*closed reference)* and 8106 (*open reference*) features at 97% percentage identity. Proceeding with the denoised dataset, we removed 28 samples due to incorrect body sites (*n* = 7), treatment prior to sampling (*n* = 11), low read counts (*n* = 5) and unknown mange status (*n* = 5). Our final dataset consisted of 125 samples collected from 12 coyotes (mange-infected = 8, uninfected = 4), 11 red foxes (mange-infected = 6, uninfected = 5) and 2 gray foxes (mange-infected = 1, uninfected = 1).

### Uninfected samples cluster by individual rather than body site

**Fig. 2:**
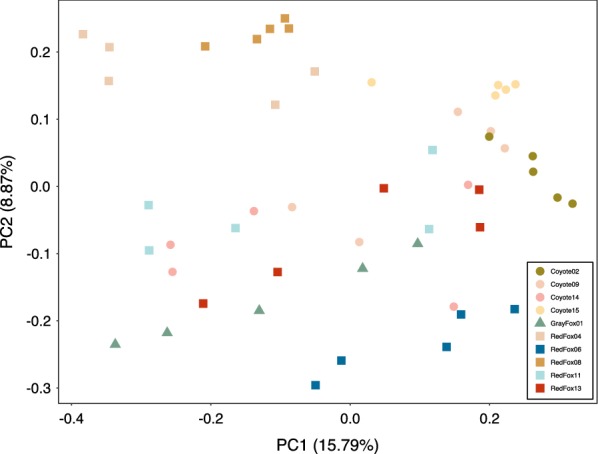
Principal coordinates analysis (PCoA) of uninfected individuals showed significant clustering by individual (PERMANOVA; *pseudo-F*_(9)_ = 2.938, *P* = 0.001) rather than body site (*pseudo-F*_(4)_ = 0.769, *P* = 0.997) using phylogeny-based unweighted UniFrac distances

Given repeated measures across individuals (*n* = 5 samples per individual) and body sites (*n* = 25 samples per body site) in the denoised dataset, we implemented principal coordinates analysis (PCoA) on uninfected samples to assess whether these factors significantly influenced beta diversity. Across all three distance measures, samples clustered by individual (PERMANOVA; Bray-Curtis, *pseudo-F*_(9)_ = 2.984, *P* = 0.001; unweighted UniFrac, *pseudo-F*_(9)_ = 2.938, *P* = 0.001; weighted UniFrac, *pseudo-F*_(9)_ = 3.470, *P* = 0.001) rather than body site (Bray-Curtis, *pseudo-F*_(4)_ = 0.781, *P* = 0.997; unweighted UniFrac, *pseudo-F*_(4)_ = 0.769, *P* = 0.997; weighted UniFrac, *pseudo-F*_(4)_ = 0.950, *P* = 0.574; Fig. 2, Additional file 2: Figure S1). We therefore grouped samples by individual in downstream analyses to control for statistical relicts of pseudoreplication. Rather than five samples per canid (i.e. one for each body site), each individual was represented by one composite sample that contained all features in their skin microbiome.

We performed significance testing for alpha and beta diversity on our composite dataset to determine which metadata categories were predictive of microbial community structure. Mange infection was consistently the variable most strongly associated with differences in alpha and beta diversity (Additional file 3: Table S2). Although sex appeared significant, further analyses showed non-independence between sex and mange status (Chi-square test, χ^2^ = 4.039, *df* = 1, *P* = 0.044), due to a disproportionate number of infected males in the dataset. Notably, test statistics calculated for sex were lower than those calculated for mange infection status (Additional file 3: Table S2). We further performed significance testing on uninfected canids of known sex to see whether male and female canids exhibited different microbial communities. In these analyses, we observed no significant differences in alpha or beta diversity between the sexes (Additional file 4: Table S3). These results were visually confirmed through PCoA (Additional file 5: Figure S2). Considered together, this evidence suggested that mange infection status, rather than sex, was the primary driver underlying differences in microbial community structure. We therefore analyzed the full composite dataset for subsequent analyses and used mange infection status as our variable of interest.

### Mange-infected canids exhibit decreased diversity and community evenness across species

**Fig. 3:**
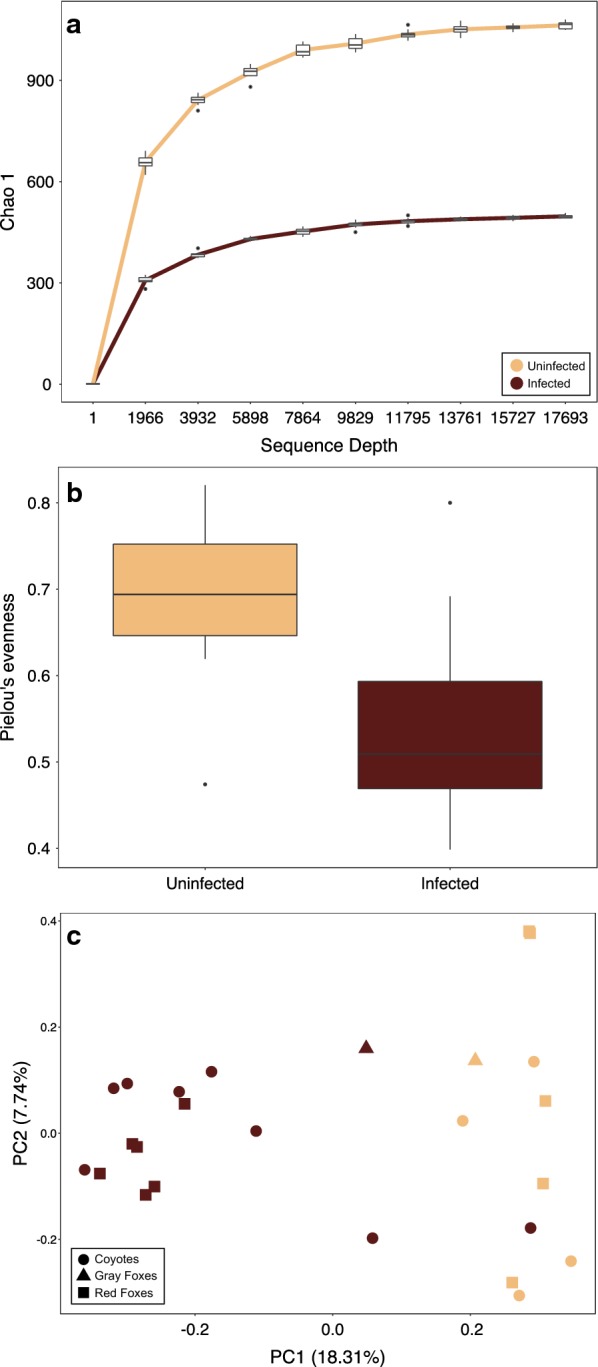
Mange-infected individuals had significantly reduced **a** species richness (Kruskal–Wallis test; Chao 1, *H* = 10.711, *P* = 0.001) and **b** evenness (Pielou’s evenness metric, *H* = 8.643, *P* = 0.003) when compared to uninfected individuals. **c** Beta diversity also differed significantly between infection groups (PERMANOVA; Bray-Curtis, *pseudo-F*_(1)_ = 3.885, *P* = 0.001)

We observed significantly reduced species richness (Kruskal–Wallis test; Chao 1 index, *H* = 10.711, *P* = 0.001; Fig. 3a) and evenness (Pielou’s evenness metric, *H* = 8.643, *P* = 0.003; Fig. 3b) in mange-infected individuals. Beta diversity similarly differed by infection group. Measures of species abundance (PERMANOVA; Bray-Curtis, *pseudo-F*_(1)_ = 3.885, *P* = 0.001; Fig. 3c), presence (unweighted UniFrac, *pseudo-F*_(1)_ = 2.211, *P* = 0.006; Additional file 6: Figure S3a), and both presence and abundance considered together (weighted UniFrac, *pseudo-F*_(1)_ = 4.398, *P* = 0.001; Additional file 6: Figure S3b) showed significant differences between mange-infected and uninfected canids. For all three measures, samples clustered by infection status along PC1, which explained 16.49–29.01% of the variation.

**Fig. 4:**
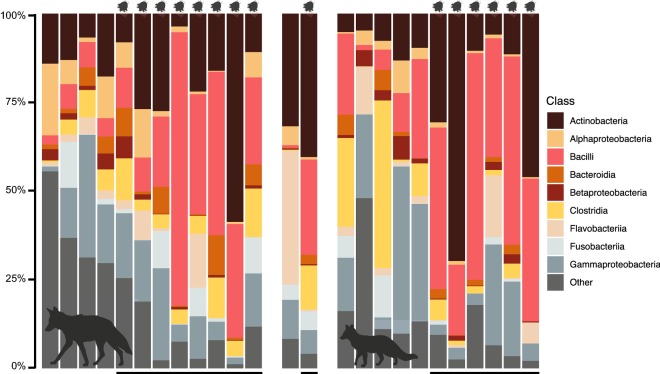
Taxonomic composition of skin microbial communities for 12 coyotes, 2 gray foxes and 11 red foxes. Black bars (bottom) and mites (top) signify individuals infected with sarcoptic mange. Figure created with BioRender

Taxonomic composition of skin microbial communities confirmed these patterns (Fig. 4). Although variation between individuals was evident, mange-infected canids exhibited higher relative abundance of Actinobacteria (mean ± standard error, SE, infected = 25.883 ± 5.183%, uninfected = 12.360 ± 2.541%) and Bacilli (mean ± SE, infected = 35.823 ± 4.898%, uninfected = 9.154 ± 2.900%), and reduced abundance of “other” taxa (mean ± SE, infected = 8.262 ± 1.883%, uninfected = 25.989 ± 5.346%). These results remained consistent even when the dataset was subdivided by species (Additional file 7: Table S4).

### Increased abundance of *Staphylococcus pseudintermedius* and *Corynebacterium* spp. with mange infection

Analysis of the composition of microbes (ANCOM) returned one taxonomic feature as consistently and significantly more abundant in mange-infected individuals: feature 3f0449c545626dd14b585e9c7b2d16f4 (*W* = 111; Additional file 8: Figure S4). NCBI BLASTn [49] search results returned high sequence similarity to *Staphylococcus pseudintermedius* (class Bacilli; Additional file 9: Table S5a). Although not statistically significant, feature e3e89166daa575e51d7a14bc65f11153 exhibited the second highest number of rejected null hypotheses (*W* = 21) and matched *Corynebacterium* spp. (class Actinobacteria; Additional file 9: Table S5b).

**Fig. 5:**
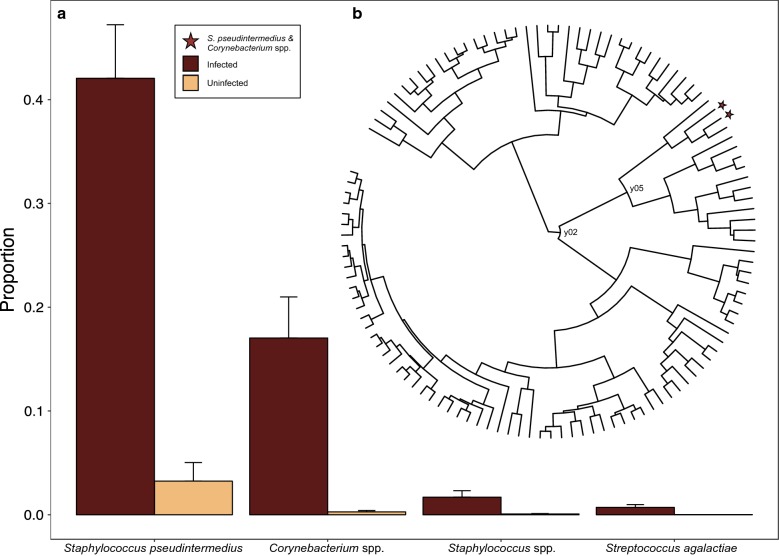
**a** Relative abundance of four taxonomic features found within gneiss balances associated with sarcoptic mange infection. *Staphylococcus pseudintermedius* and *Corynebacterium* spp. exhibited the largest differences between infection groups, with *Staphylococcus* spp. and *Streptococcus agalactiae* clustered with these taxa in the **b** hierarchy relating all features through correlation clustering

Given the strong effect of mange infection on alpha and beta diversity, we constructed a simple OLS regression model using mange infection status and gneiss balances as the variables of interest. This model explained 9.40% of the variation observed, and returned two statistically significant balances that contained features with increased taxonomic abundance in mange-infected individuals: y02 and y05 (both *P* = 0.013; Fig. 5). After visualizing the tree hierarchy through the Interactive Tree of Life (iTOL) v.3 online tool [50], we found that balance y05 was nested within y02. As a result, both balances pointed towards the same signal: increased proportion of features 3f0449c545626dd14b585e9c7b2d16f4 (mean ± SE, infected = 0.421 ± 0.052, uninfected = 0.032 ± 0.018) and e3e89166daa575e51d7a14bc65f11153 (mean ± SE, infected = 0.170 ± 0.040, uninfected = 0.003 ± 0.001) in mange-infected individuals (Fig. 5a). These features were previously identified as *S. pseudintermedius* and *Corynebacterium* spp. using NCBI BLASTn, and were clustered with two additional features in the dendrogram relating all taxa: features c2d41dc0a7b8eaedcf4697512aee4427 (identified as *Staphylococcus* spp.) and 22a5bce17370d6c495f5e83232650ec7 (identified as *Streptococcus agalactiae*; Fig. 5b). These additional features exhibited higher proportions in infected canids compared to uninfected individuals (*Staphylococcus* spp. mean ± SE, infected = 0.017 ± 0.006, uninfected = 0.001 ± 0.000; *S. agalactiae* mean ± SE, infected = 0.007 ± 0.003, uninfected < 0.001 ± 0.000). Although balance y78 was also statistically significant (*P* = 0.024), its proportions only marginally differed between infection groups, with increased abundance of its component taxa found in uninfected canids.

## Discussion

Sarcoptic mange is among the most widespread diseases affecting mammals on a global scale. Despite recognition since antiquity [2], mange is considered a neglected disease, as there remain numerous questions about its pathology in free ranging wildlife [5]. The interplay between mites and microbes at the skin barrier is one such question, given increasing recognition of the importance of host-associated microbiomes in wildlife health and disease [51–53].

We characterized the skin microbiome of mange-infected and uninfected canids in three North American species: coyotes, red foxes and gray foxes. Across species, we observed remarkably consistent signatures of mange infection that included reduced diversity, shifted community composition and increased proportion of *S. pseudintermedius* and *Corynebacterium* spp. Although samples derived from different species sampled in different states, infection status was the primary driver of microbial community structure in terms of species richness, evenness, presence and relative abundance.

Commensal microbial communities are shaped by a complex milieu of genetic and environmental factors [54, 55]. Although inter-individual variation is pervasive, the host-associated microbiome is thought to exhibit phylosymbiosis between microbes and their hosts over evolutionary timescales [56, 57]. In a study of small mammals spanning six genera, for example, species identity exerted a far stronger effect on microbial community structure than did local habitat [58]. We therefore anticipated divergence between the skin microbiome of the three focal species, as coyotes, red foxes and gray foxes are in different genera within Canidae. Counter to this expectation, we found minimal differences between skin microbial communities across species, sampling locations, years, sexes and ages. Instead, mange infection status was the primary factor associated with microbial community structure within our dataset. This suggested two primary hypotheses. The first posits that shared evolutionary history and contemporary ecology of these species leads to similar skin microbiomes, as seen in gut microbial communities across families within class Mammalia [59]. The second contends that mange infection alters community composition consistently and dramatically across species, thereby blurring inter-genus distinctions within our relatively small sample set.

Results from this study primarily supported the second hypothesis, although it is likely that evolutionary history, contemporary ecology and mange infection all influenced the observed patterns of microbial diversity. Within the broader context of microbes and mange, reduced microbial variation and increased abundance of opportunistic pathogens is consistent with humans infected with *S. scabiei* var. *hominis* [60, 61], pigs experimentally infected with *S. scabiei* var. *suis* [28], Santa Catalina island foxes (*Urocyon littoralis catalinae*) infected with *Otodectes cynotis* ear mites [36], and domestic dogs and humans exhibiting allergic skin disorders [31–34]. Although the identity of opportunistic pathogens varied by host species, *Staphylococcus* spp. and *Streptococcus* spp. were commonly reported. Mite presence may even facilitate these secondary bacterial infections by secreting proteins that inhibit the mammalian complement system, which is known to be a key player in the immune response against mite and bacterial infections [26, 27, 62, 63]. Mite burrows and host lesions may therefore provide ideal environments for opportunistic pathogens to proliferate.

The primary microbial taxa associated with mange infection in this study included *S. pseudintermedius* and *Corynebacterium* spp., with *S. agalactiae* and other *Staphylococcus* spp. marginally differing in abundance. Both humans and pigs infected with *S. scabiei* exhibited increased proportion of *S. aureus* [28, 60], with *S. pseudintermedius* reported in island foxes infected with ear mites [36]. These analogous results present compelling evidence that mite infection is associated with *Staphylococcus* spp. proliferation across host taxa. Further, increased abundance of *S. pseudintermedius* across four canid species infected with *S. scabiei* (coyotes, red foxes and gray foxes) and *O. cynotis* (island foxes [36]) mites suggests that it is an important bacterial taxon within Canidae.

A common canid commensal [64], *S. pseudintermedius* becomes an opportunistic pathogen when the skin microbiome is disrupted by allergic skin disease, infection or surgery [65–67]. Resultant biofilms can lead to chronic inflammation in domestic dogs, cats (*Felis catus*) and, to a lesser extent, humans [68], with antibiotic resistant strains emerging across veterinary and medical hospitals [69, 70].

Although less commonly reported across host species, *Corynebacterium* spp. was detected in skin crusts and *S. scabiei* mites isolated from pigs with severe mange [28]. Similar bacteria were also isolated from the gastrointestinal tracts of hematophagous arthropods, such as triatomes (*Triatoma infestans* [71]) and three species of ticks (*Ixodes ricinus*, *Dermacentor reticulatus* and *Haemaphysalis concinna* [72]). This evidence suggests that *Corynebacterium* spp. may derive from mite bodies, secretions or frass deposited at the site of infection, in addition to canid commensal communities. As with *S. pseudintermedius*, these bacteria likely benefit from mite inhibition of mammalian complement.

## Conclusions

In the present study, we reported microbial dysbiosis associated with sarcoptic mange infection in three species of North American canids: coyotes, red foxes and gray foxes. Across species, mange was consistently characterized by decreased microbial diversity, altered community composition and increased proportion of opportunistic bacterial infections comprised of *S. pseudintermedius* and *Corynebacterium* spp. These additional insights into the pathogenesis of sarcoptic mange may enable novel management of wildlife affected *in situ* and *ex situ* [73]. Regarding treatment, acaricides may possess harmful side effects for individuals and the environment, with drug resistance observed in some *S. scabiei* lineages and their concomitant bacterial infections [1, 6, 70, 74]. It may become critical to pursue novel avenues of treatment, such as a combination of acaricides and anti- or probiotic therapies, to improve therapeutic outcomes for infected individuals. Insights into mite microbiomes may further provide means of mite control if these communities can be manipulated [71]. Given the ubiquity of this disease and its capacity to infect humans, domestic animals and wildlife, sarcoptic mange presents an ideal candidate for adopting a “One Health” perspective when mitigating its negative effects [5]. Mammalian hosts can be intricately coupled, enabling interspecies transmission when diseased animals approach human settlements in search of resources or shelter, as seen in mange-infected coyotes [75, 76] and red foxes [77]. Although public health concerns are minor due to the lesser severity of zoonotic mange, interspecies transmission between widespread and at-risk species can pose a conservation risk. Thus, identifying consistent drivers of morbidity, such as altered microbial communities, can enable better prediction and mitigation of mange dynamics across host systems.

## Supplementary information

**Additional file 1: Table S1.** Sample metadata and sequence processing statistics for each stage of the *dada2* denoising pipeline implemented in *QIIME2* v.2019.4.

**Additional file 2: Figure S1.** Principal coordinates analyses of uninfected individuals showed significant clustering by individual rather than body site using both **a** Bray-Curtis and **b** phylogeny-based weighted UniFrac distances.

**Additional file 3: Table S2.** Results from alpha (Kruskal–Wallis test) and beta (PERMANOVA) diversity significance tests performed on the composite dataset.

**Additional file 4: Table S3.** Results from alpha (Kruskal–Wallis test) and beta (PERMANOVA) diversity significance tests performed on uninfected canids with known sex.

**Additional file 5: Figure S2.** Principal coordinates analysis of **a** uninfected individuals (five samples per canid) using phylogeny-based unweighted UniFrac distances and **b** all individuals (one composite sample per canid) using Bray-Curtis dissimilarity index show minimal evidence of clustering by sex (females, no outline; males, black outline; unknown, gray outline).

**Additional file 6: Figure S3.** Principal coordinates analysis showed significant differences between infection groups using both **a** unweighted and **b** weighted UniFrac distances.

**Additional file 7: Table S4.** Relative abundance (mean and standard error) of taxonomic classes in canids grouped by species and infection status. Taxonomic classes include: Bacilli, Actinobacteria, Gammaproteobacteria, Clostridia, Flavobacteriia, Alphaproteobacteria, Fusobacteriia, Bacteroidia, Betaproteobacteria, and other taxa.

**Additional file 8: Figure S4**. Analysis of the composition of microbes returned one taxonomic feature as consistently and significantly associated with mite infection status: *Staphylococcus pseudintermedius* (indicated with a red star). Three additional taxa that commonly co-occurred with *S. pseudintermedius* included *Corynebacterium* spp. (pink star), *Streptococcus agalactiae* (red circle) and *Staphylococcus* spp. (pink circle).

**Additional file 9: Table S5.** NCBI BLASTn results for the two features exhibiting increased relative abundance in mange-infected individuals: **a** 3f0449c545626dd14b585e9c7b2d16f4 (class Bacilli) and **b** e3e89166daa575e51d7a14bc65f11153 (class Actinobacteria).

## Data Availability

All sequencing data analyzed in the present study is publically available through the NCBI Sequence Read Archive under BioProject PRJNA562927. Demultiplexed, paired-end fastq files are available for each sample (BioSamples SAMN12659808 to SAMN12659960) with SRA accession numbers: SRR10044131 to SRR10044283. Sample metadata is publically available through Additional file 1: Table S1.

## Abbreviations

ANCOM: analysis of composition of microbes
BLASTn: basic local alignment search tool for nucleotides
iTOL: Interactive Tree of Life
NCBI: National Center for Biotechnology Information
OLS: ordinary least squares
OTU: operational taxonomic unit
PC1: principal coordinate one
PCoA: principal coordinates analysis
PCR: polymerase chain reaction
PERMANOVA: multivariate analysis of variance with permutation
rRNA: ribosomal RNA
V4: hypervariable region 4
