# Supplementary figures and images for: Of microbes and mange: consistent changes in the skin microbiome of three canid species infected with Sarcoptes scabiei mites
Source: Parasit Vectors. 2019 Oct 16;12:488. doi: 10.1186/s13071-019-3724-0 (PMC6796464; doi:10.1186/s13071-019-3724-0)

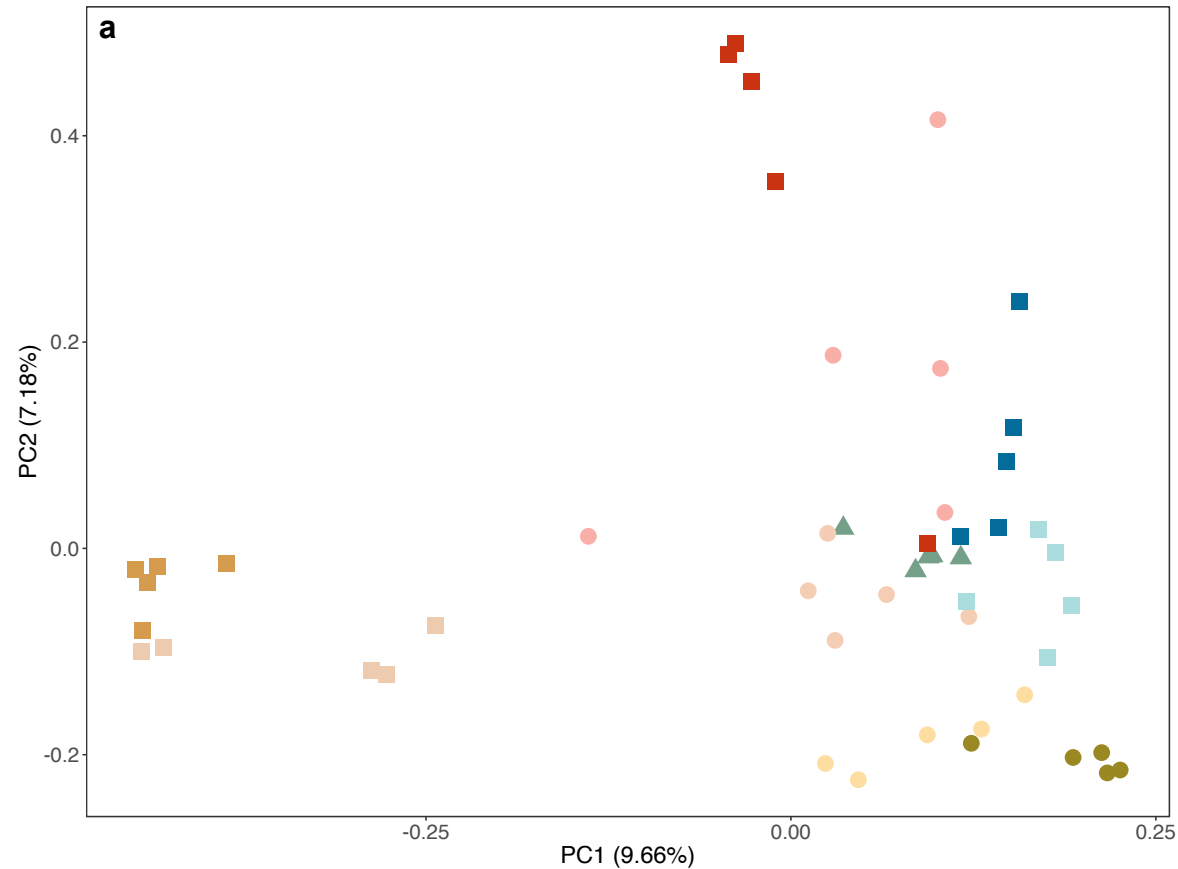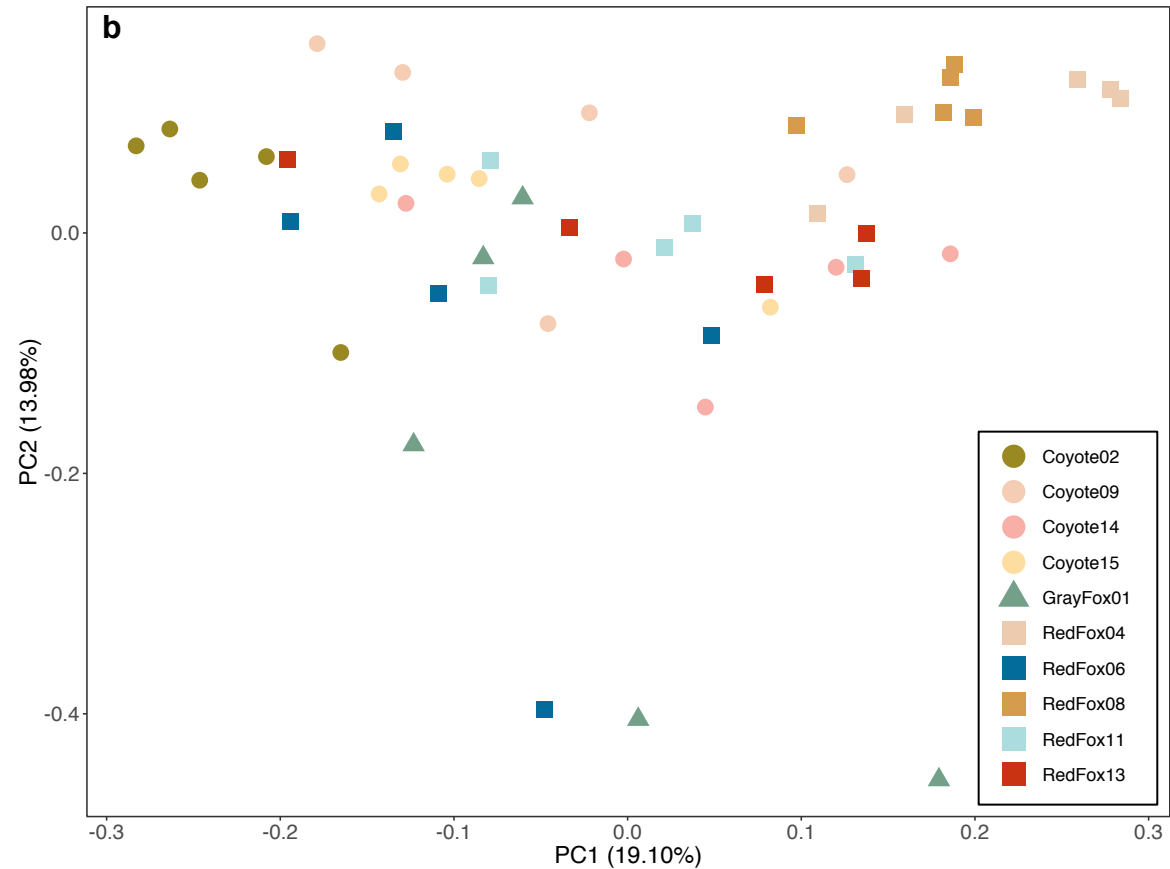

Supplement: Supplementary file 2 — Additional file 2: Figure S1. Principal coordinates analyses of uninfected individuals showed significant clustering by individual rather than body site using both a Bray-Curtis and b phylogeny-based weighted UniFrac distances. [file 13071_2019_3724_MOESM2_ESM.pdf]

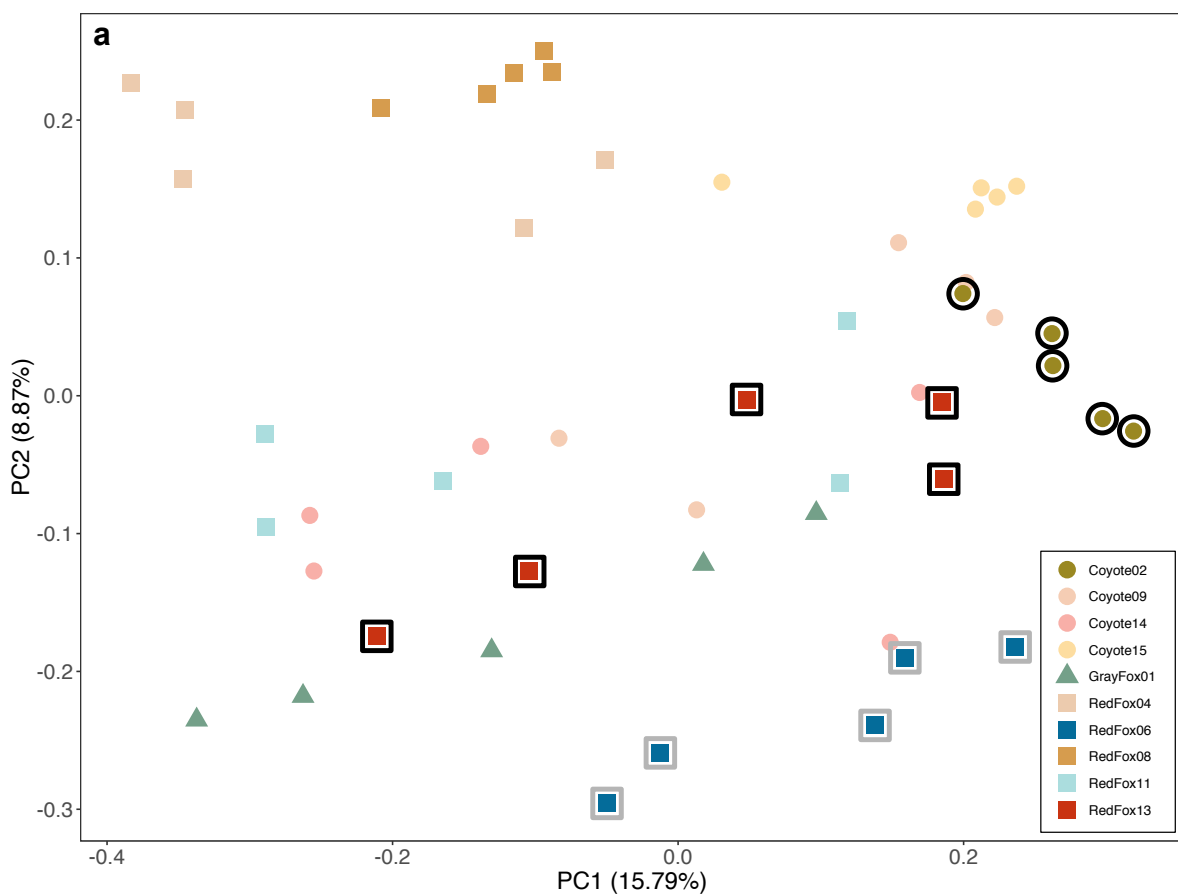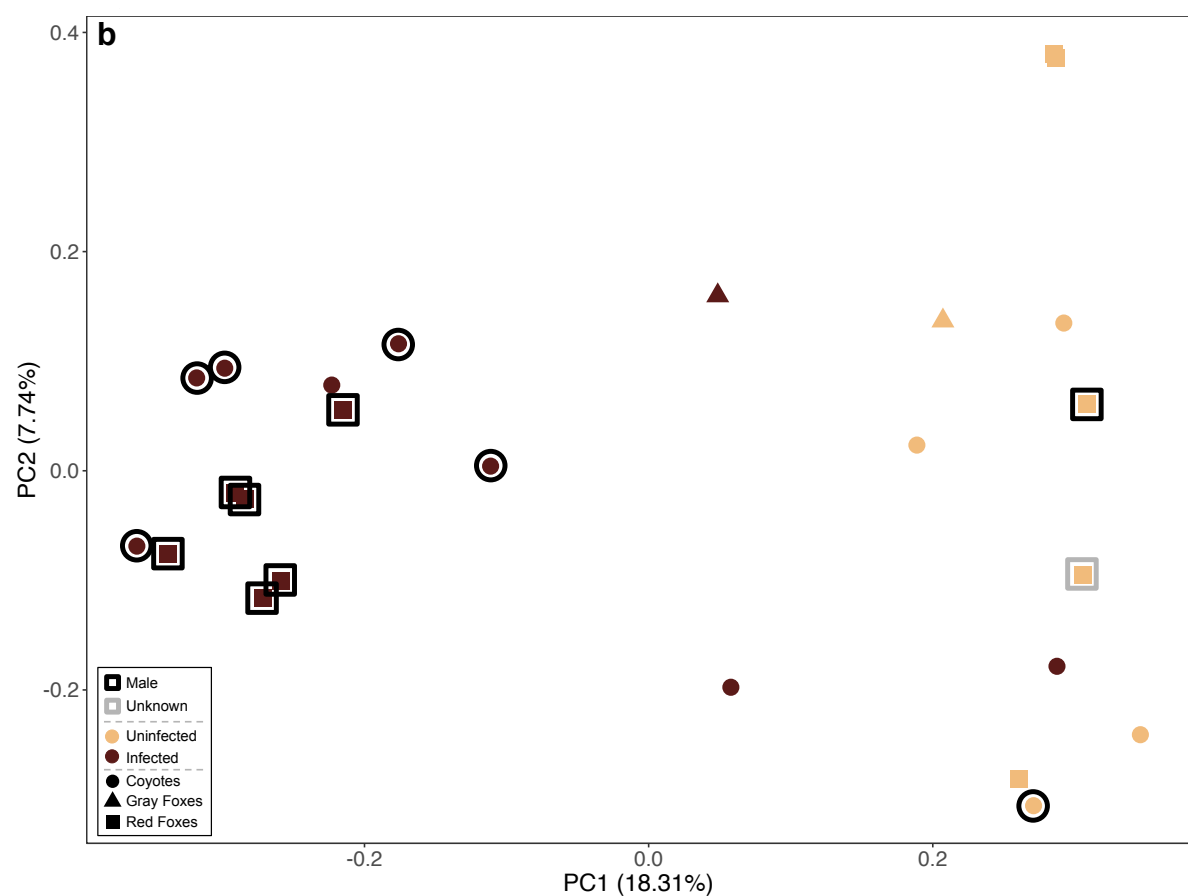

Supplement: Supplementary file 5 — Additional file 5: Figure S2. Principal coordinates analysis of a uninfected individuals (five samples per canid) using phylogeny-based unweighted UniFrac distances and b all individuals (one composite sample per canid) using Bray-Curtis dissimilarity index show minimal evidence of clustering by sex (females, no outline; males, black outline; unknown, gray outline). [file 13071_2019_3724_MOESM5_ESM.pdf]

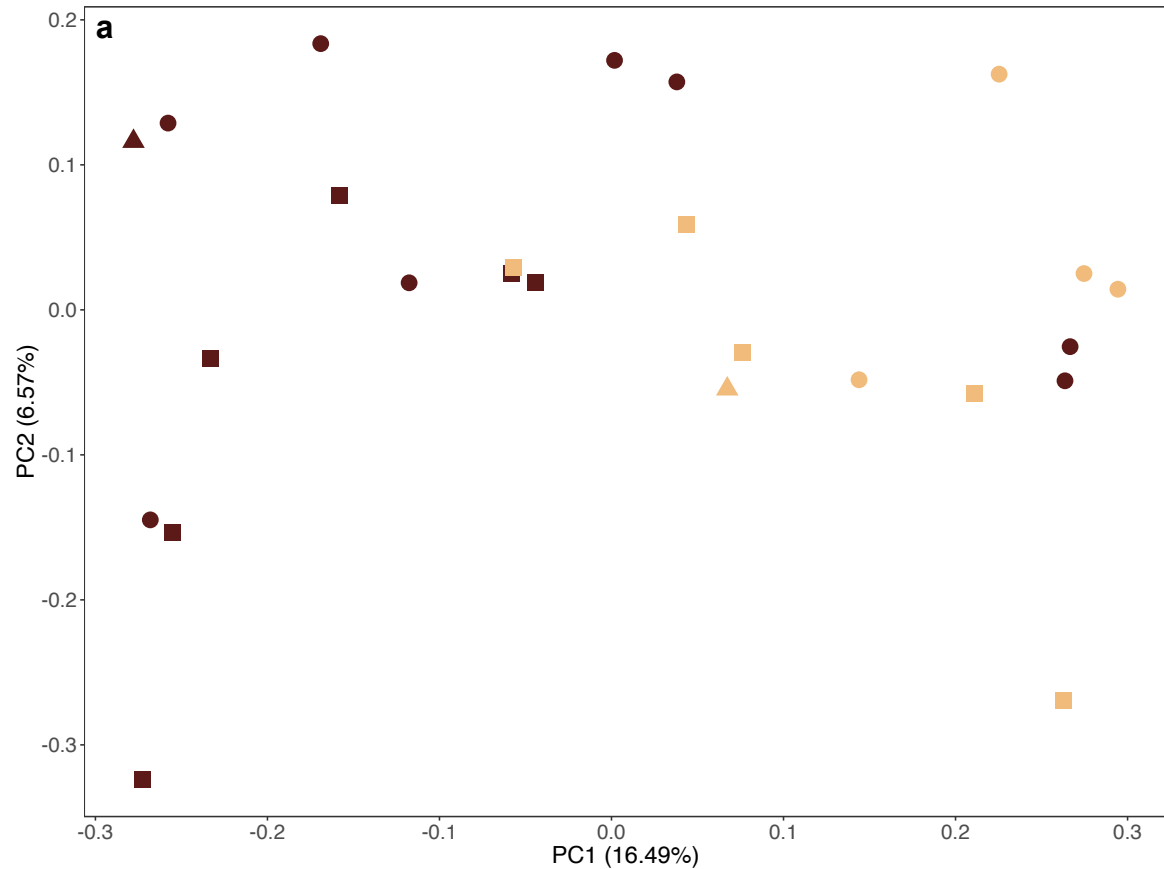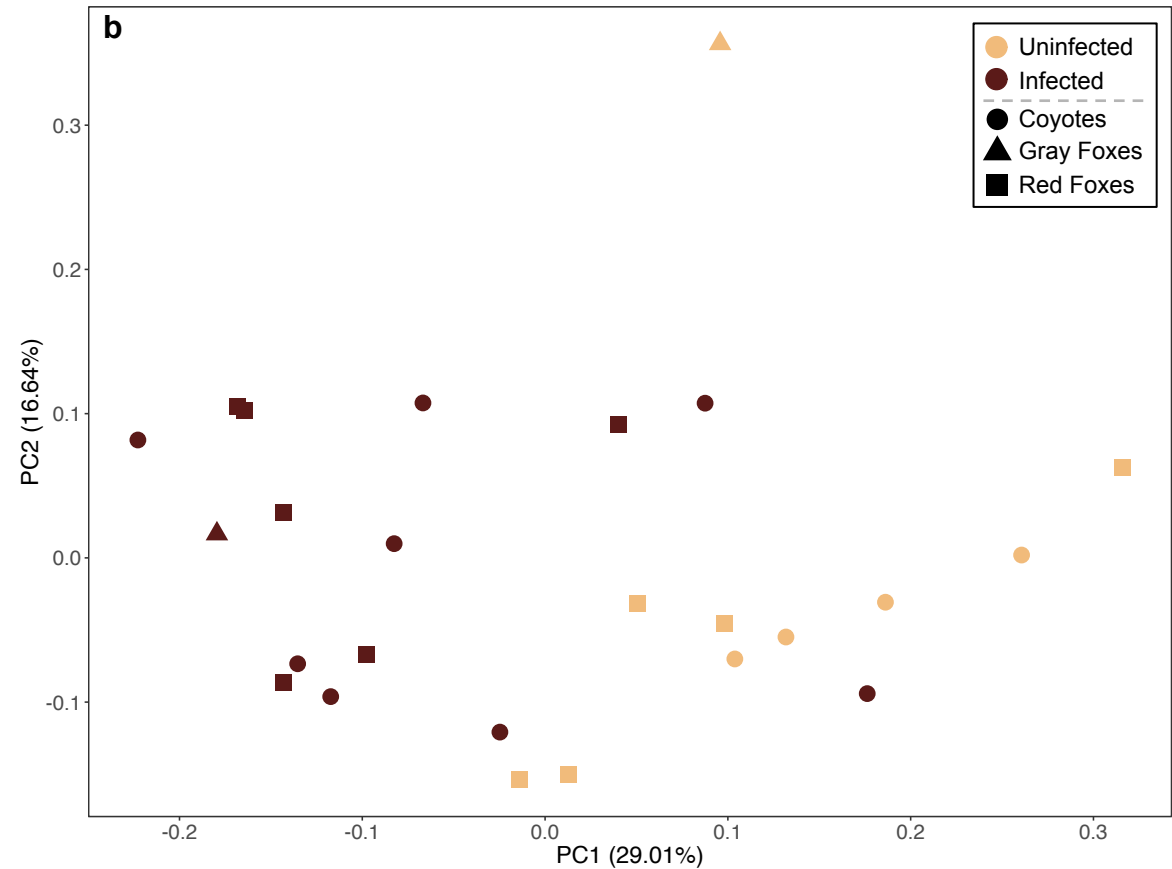

Supplement: Supplementary file 6 — Additional file 6: Figure S3. Principal coordinates analysis showed significant differences between infection groups using both a unweighted and b weighted UniFrac distances. [file 13071_2019_3724_MOESM6_ESM.pdf]

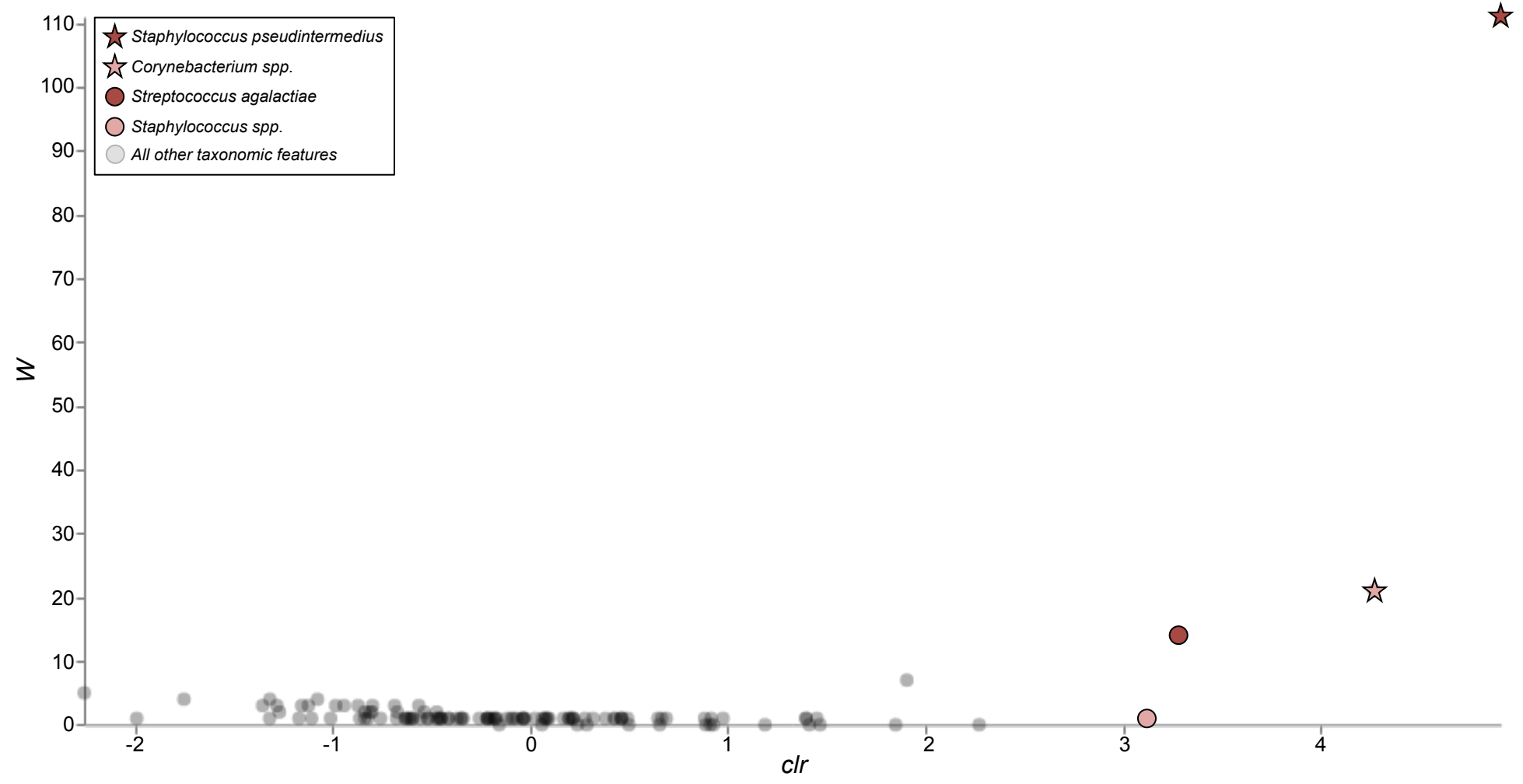

Supplement: Supplementary file 8 — Additional file 8: Figure S4. Analysis of the composition of microbes returned one taxonomic feature as consistently and significantly associated with mite infection status: Staphylococcus pseudintermedius (indicated with a red star). Three additional taxa that commonly co-occurred with S. pseudintermedius included Corynebacterium spp. (pink star), Streptococcus agalactiae (red circle) and Staphylococcus spp. (pink circle). [file 13071_2019_3724_MOESM8_ESM.pdf]
